# Systematic Analysis and Biomarker Study for Alzheimer’s Disease

**DOI:** 10.1038/s41598-018-35789-3

**Published:** 2018-11-26

**Authors:** Xinzhong Li, Haiyan Wang, Jintao Long, Genhua Pan, Taigang He, Oleg Anichtchik, Robert Belshaw, Diego Albani, Paul Edison, Elaine K Green, James Scott

**Affiliations:** 1 Plymouth University Faculty of Medicine and Dentistry, Drake Circus, Plymouth, PL4 8AA UK; 2 Department of Methodology, London School of Economics and Political Science, Houghton St, London, WC2A 2AE UK; 3 School of Computing Electronics and Mathematics, Plymouth University, Drake Circus, Plymouth, PL4 8AA UK; 4 Molecular and Clinical Sciences Research Institute, St George’s, University of London, Cranmer Terrace, London, SW17 0RE UK; 5 Department of Neuroscience, IRCCS - Istituto di Ricerche Farmacologiche “Mario Negri” Via La Masa 19, 20156 Milan, Italy; 6 Department of Medicine, Imperial College London, Du Cane Road, London, W12 0NN UK

## Abstract

Revealing the relationship between dysfunctional genes in blood and brain tissues from patients with Alzheimer’s Disease (AD) will help us to understand the pathology of this disease. In this study, we conducted the first such large systematic analysis to identify differentially expressed genes (DEGs) in blood samples from 245 AD cases, 143 mild cognitive impairment (MCI) cases, and 182 healthy control subjects, and then compare these with DEGs in brain samples. We evaluated our findings using two independent AD blood datasets and performed a gene-based genome-wide association study to identify potential novel risk genes. We identified 789 and 998 DEGs common to both blood and brain of AD and MCI subjects respectively, over 77% of which had the same regulation directions across tissues and disease status, including the known *ABCA7*, and the novel *TYK2* and *TCIRG1*. A machine learning classification model containing *NDUFA1*, *MRPL51, and RPL36AL*, implicating mitochondrial and ribosomal function, was discovered which discriminated between AD patients and controls with 85.9% of area under the curve and 78.1% accuracy (sensitivity = 77.6%, specificity = 78.9%). Moreover, our findings strongly suggest that mitochondrial dysfunction, NF-κB signalling and iNOS signalling are important dysregulated pathways in AD pathogenesis.

## Introduction

Alzheimer’s Disease (AD) accounts for 60–80% of all dementia cases (http://www.alz.org). By 2050, the number of people with AD is predicted to increase from 5.4 million to between 11 and 16 million in the U.S alone, and it is estimated that dementia will cost $2 trillion by 2030 worldwide (http://www.alz.org). Despite these alarming numbers, there is no effective strategy to identify pre-symptomatic disease, which might be the only stage of the disease’s trajectory where we could intervene.

Genomics and genetics approaches have made great progress in revealing the mechanisms underlying Alzheimer’s disease. Genome-wide association studies (GWAS) and meta-analyses have identified 23 statistically significant AD associated genes^1^. In total 39 AD risk genes have been identified so far^2,3^, including *APOE, APP, TRIP4, ABCA7, and SORL1*. These genes highlight the importance of various pathways involved in AD, such as immune response and inflammation, cell migration, lipid transport and endocytosis, hippocampal synaptic function and other cell regulatory processes, along with the role of tau and amyloid protein^1^. The majority of published gene expression studies have been performed using post-mortem brain tissues and as such have focused on the later stages of the advanced disease^4–7^. A key need is to explore how these changes in the brain relate to changes in the blood. The availability of gene expression data from brain tissue and blood cells now make it possible to compare these two tissues, and holds out the possibility of identifying in the blood a panel of predictive biomarkers that are mechanistically associated with this disease in the brain.

No single biomarker, e.g., gene or protein, is likely be a reliable biomarker for early AD. Previous studies have therefore used machine learning (ML) to build multi-biomarker models for clinical diagnosis and prediction of AD based on measurement of RNA, protein, and lipid levels in blood samples^8,9^. Support Vector Machine (SVM) and random forest (RF) models have proved predictive in distinguishing between cognitively normal, mild cognitive impairment (MCI), i.e. prodromal AD, and subjects with AD using gene expression^10,11^ and blood analytes^12^. Pathway-based classification approaches for blood-based AD diagnosis have also been used, with age and APOE4 status of the subjects included as covariates (these are the two known biggest risk factors^13^). However, as these studies were performed on a variety of platforms with different initial feature sizes and relatively small sample size, very few potential biomarkers have so far been identified or replicated in larger cohort study^14^.

Our study has two parts. The first was a system analysis to identify differentially expressed genes (DEGs) and pathways in a large-scale human blood dataset, and integrate these with results from brain tissue to comprehensively explore the correlations between blood and brain. The second part was to apply ML techniques to identify a panel of potential predictive biomarkers in the blood, and to see whether gene expression in the blood can be used as a biomarker for AD diagnosis.

## Methods

### Microarray gene expression profile in human blood

Two independent human whole blood normalized mRNA gene expression datasets were downloaded from GEO (http://www.ncbi.nlm.nih.gov/geo/): GSE63060 and GSE63061 from the AddNeuroMed Cohort^15^. We merged these two normalized datasets (generated by different Illumina platforms) using the inSilicoMerging R package^16^, and then extracted 143 patients with AD, 77 MCIs and 104 controls subjects (CTL) from GSE63060; 102 patients with AD, 65 MCIs and 78 CTLs from GSE63061 with Western European and Caucasian ethnicity respectively. Probesets without annotation (Entrez_Gene_ID) were filtered out, which left 22756 probesets corresponding to 16928 unique genes. The limma R package^17^ was then applied and adjusted by age and gender to identify DEGs (a) between AD patients and CTLs, (b) between MCI patients and CTL groups, and (c) between AD and MCI patients. These comparisons were carried out in the two GEO datasets and in the merged one (referred to as the merged discovery dataset) separately. We focused on this merged discovery dataset for downstream analysis with the Benjamini-Hochberg adjusted p-value, i.e. BH.pval of 0.01 used as the significance level for DEG identification.

In order to evaluate the DEGs identified in our above discovery dataset, two additional datasets were downloaded for analysis. Firstly, the whole blood gene expression dataset (GSE6613) was download from GEO. The Affymetrix U133A CEL profiles were normalized by RMA^18^ method implemented in affy R package. Probesets were filtered out if (1) they were not annotated or were multiply annotated; or (2) they were present in less than 10 percent of the samples as determined by applying the MAS5 present/absent call algorithm (affy R package). DEGs were identified by applying limma with age and gender adjusting. Nominal pval < 0.01 was used for significance because we observed that no DEG could pass multiple testing (BH.pval > 0.05, see discussion section). This dataset includes samples for AD, MCI, CTL, as well as Parkinson disease (PD). We excluded PD samples after data normalisation.

The second evaluation blood gene expression dataset was downloaded from the Alzheimer’s Disease Neuroimaging Initiative website (ADNI, http://www.adni-info.org/). The ADNI was launched in 2003 as a public-private partnership led by Principal Investigator Michael W. Weiner, MD. The primary goal of ADNI has been to test whether serial magnetic resonance imaging (MRI), positron emission tomography (PET), other biological markers and clinical and neuropsychological assessment can be combined to measure the progression of MCI and early AD. In our study, we focused on the ADNI2 Caucasian population with disease status according to baseline diagnosis. This cohort has APOE4 information for each individual participant. Limma was applied to each APOE4 group (APOE4 = 0, APOE4 = 1, APOE4 = 2), adjusting for age, gender, RIN, RNA purity ratio A_260/280_ and A_260/230_ separately to detect DEGs between patients with AD and CTL, early MCI (EMCI) and CTL, late-MCI (LMCI) and CTL. A nominal p-value of <0.01 was used for significance since no DEG could pass multiple testing (see discussion section). We present results on the APOE4 = 1 group because there were similar numbers of cases for each disease status in this group, but very few AD cases in the other two APOE4 groups.

### Microarray gene expression profile in human brain

The GSE84422 dataset includes human post-mortem brain samples taken from 19 brain regions for an AD study^6^. The cohort used is totally independent to the above blood cohorts. Gene expression profiles of 17 brain regions were generated by both Affymetrix U133A and U133B platforms, and profiles for other two regions were generated by the U133plus2 platform. We processed the raw CEL files as above, identified DEGs for each platform separately adjusted by age, gender, post-mortem interval (PMI) and pH values using limma, as applied in the original study^6^, and merged them together afterwards to obtain 19 lists of DEGs. Nominal pval < 0.01 was applied for significance, again since no DEG could pass multiple testing (i.e. BH.pval > 0.05). We only analysed definite AD and CTLs in the Caucasian ethnic group. Supplementary Table 1 indicates the sample size in each comparison group including the cases for blood datasets.

To clarify, within our study, DEGs either refer to array probesets, when we discuss DEGs within the same data cohorts, or unique genes (Entrez_Gene_ID), when we compare results from different cohorts for blood and brain.

### Pathway analysis for DEGs

We performed pathway analysis on the identified DEGs using commercial Ingenuity® Pathway Analysis (IPA®, QIAGEN Redwood City, www.qiagen.com/ingenuity) software. We chose as significant those canonical pathways with BH.pval < 0.01.

### Gene-based Analysis of GWAS data

The International Genomics of Alzheimer’s Project (IGAP) Consortium reported a large-scale of AD GWAS dataset^1^. The gene-based analysis tool MAGMA^19^ was applied to the IGAP stage 1 whole genome summary statistics (including 17,008 AD and 37,154 CTLs), with the 1000 genomes European reference panel used to perform the joint SNP gene-based GWAS study. We searched for single-nucleotide polymorphisms (SNPs) within 20 kb up/downstream of each gene (NCBI37.3). Two significance levels were applied, nominal pval < 0.01 and Bonferroni BF.pval < 0.05 to identify significant genes in GWAS, which we refer to as MAGMA genes. The qvalue package in R was also applied.

### Biomarker discovery by machine learning

We attempted to identify blood biomarkers and classification models trained/learned from the GSE63060 dataset and tested in GSE63061, and vice versa. Data were adjusted for age and gender by a robust regression model (applying the rlm function in MASS R package); the model residual was further centred and scaled to a mean of zero and standard variation of one across all subjects in each dataset for those common probesets. We used the least absolute shrinkage and selection operator (LASSO) regression feature selection method^20^, implemented in the glmnet R package, to investigate the prediction performance of different ML approaches, including SVM, RF and logistic Ridge Regression (RR) models with a voting strategy to detect optimal biomarkers and classification models to discriminate AD patients from control subjects. The voting strategy of majority outcomes from the above three ML algorithms was applied to determine the final predictive outcome. The LASSO approach shrank most of the coefficients of variables that have no or less discriminatory power to zero, while variables with non-zero coefficients remained in the final LASSO model representing the joint discriminatory power to separate patients with AD and controls subjects^21^. An optimal penalty factor lambda was tuned during the cross-validation process. We repeated such LASSO regression with 5-fold cross-validation (CV) 100 times, and the subset of features with the best CV area under the curve (AUC) value for receiver operating characteristic (ROC), or most frequently selected on the training dataset, was kept as the selected biomarker panel (feature set). However, if the number of variables selected was less than two, then the feature set with sub-optimal AUC would be selected. Feature set selected by LASSO initially started from the full feature pool, i.e., 22756 common probesets between GSE63060 and GSE63061. For SVM and RF, we used the default setting when calculating the predict accuracy. For RR, we calculated the optimal cut-off from training with optimal AUC and accuracy, and then applied this cut-off to prediction in testing. Prediction performances of the classifiers were evaluated by AUC, test accuracy (ACC), sensitivity (Sens), and specificity (Spec). For comparison, the area under precision-recall curve (AUPR) were calculated as well using PRROC R package. ROC curves were plotted using the ROCR R package^22^. All this work was conducted by in-house R programs.

## Results

### Differentially expressed genes in blood were also found in the brain

DEGs identified in the blood merged discovery dataset included 4980 (4276 unique genes) and 6739 (5746 unique genes) probesets for AD and MCI respectively (Supplementary Fig. 1), with 4158 common probesets representing 3601 unique genes. Only 82 probesets (76 unique genes) were identified as DEGs comparing AD to MCI, and only three of these 82 were DEGs in both AD and MCI (Supplementary Fig. 2 and Supplementary Table 2). It was observed that DEGs in AD (AD-DEGs) are likely to be DEGs in MCI (MCI-DEGs) with a highly significant enrichment (OR = 29.1, 95%CI 26.7–31.7, pval < 1.0E-16, Fisher test). In addition, those common DEGs shared the same regulatory directions in both AD and MCI (Supplementary Fig. 2), i.e., 2018 of them were up-regulated both in AD and MCI, while 2140 of them were down-regulated in both AD and MCI. Moreover, those common DEGs have larger changes in MCI compared to controls than in AD compared to controls (wilcox.test p.val < 2.2e-16). It is interesting that this observation holds for all the DEGs in MCI and AD (wilcox.test p.val < 4.06e-7). In addition, both AD-DEGs and MCI-DEGs in blood were significantly associated (absolute Pearson correlation |r| > 0.5) with Braak pathological stage (OR > 1.4, pval < 8.62E-14) or frontal atrophy (OR > 1.2, pval < 8.4E-06) in the brain subjects with AD when mapped to the data in Zhang’s brain study^4^ (Supplementary Fig. 3). Furthermore, 789 AD-DEGs in blood were also DEGs identified by our previous meta-analysis in brain prefrontal cortex (PFC) region^7^ with significant enrichment (OR = 1.48, 95%CI 1.34–1.62, pval < 6.28E-16), and 77.9% of them showed the same regulation direction between blood and brain (pval < 2.2E-16, sign test). Similarly, we observed that 998 MCI-DEGs in blood are also DEGs in the brain of AD patients with significant enrichment (OR = 1.39, 95%CI 1.27–1.51, pval = 4.90E-13). Peters *et al*. recently identified 1497 genes as being differentially expressed with chronological age^23^, and we observed that AD-DEGs or MCI-DEGs in blood were likely to be ageing-associated genes (OR > 2.00, pval < 2.93E-36 for both, Supplementary Fig. 3). AD-DEGs in brain PFC region^7^ were also enriched with these ageing-associated genes, although with a slightly lower level of enrichment (OR = 1.8, 95%CI 1.6–2.1, pval < 2.2E-16).

Table 1 lists the top 20 DEGs common to both AD and MCI, the top 10 AD-only DEGs, and the top 10 MCI-only DEGs in blood (see Supplementary Table 2 for the whole list).

**Table 1:** The top DEGs in blood and their relationships with AD brain.

Entrez ID	Symbol	Blood AD FC	Blood AD BH.pval	Blood MCI FC	Blood MCI BH.pval	Brain AD FC	Brain AD meta pval	Brain AD BF.pval	Brain BraakR	Brain AtrophyR
**Top 10 up and top 10 down AD DEGs in blood. Both are MCI DEGs in blood as well**										
51258	MRPL51	0.71	3.04E-44	0.74	1.49E-26	0.99	8.27E-01	1		
4694	NDUFA1	0.52	2.34E-43	0.56	1.40E-27	0.86	2.49E-03	1		−0.46
6166	RPL36AL	0.63	7.18E-40	0.67	1.39E-20	1.05	2.19E-01	1		
4725	NDUFS5	0.58	5.22E-38	0.63	4.58E-23	0.85	1.08E-03	1		
401206	LOC401206	0.65	1.94E-36	0.71	2.56E-20					
646200	LOC646200	0.56	1.94E-36	0.60	4.31E-23					
6230	RPS25	0.64	1.49E-35	0.68	3.24E-21	1.16	2.82E-07	6.64E-03		
521	ATP5I	0.64	6.06E-35	0.67	1.03E-22	1.02	9.51E-02	1		
10063	COX17	0.73	1.29E-34	0.75	2.82E-23	0.97	3.02E-02	1		
7388	UQCRH	0.62	1.19E-33	0.66	5.82E-21	0.88	2.87E-03	1	−0.63	−0.50
10312	TCIRG1	1.26	9.61E-21	1.24	8.90E-15	1.29	1.49E-13	3.51E-09	0.67	0.54
6645	SNTB2	1.18	1.88E-20	1.20	1.06E-19	1.12	2.36E-03	1	0.52	0.46
7297	TYK2	1.22	3.69E-20	1.21	5.48E-16	1.13	1.57E-09	3.68E-05	0.67	0.51
153222	C5orf41	1.20	6.74E-18	1.12	1.97E-06	1.12	3.47E-06	0.08	0.56	
9931	HELZ	1.12	2.55E-17	1.12	8.45E-15	1.02	5.05E-04	1		
730994	LOC730994	1.19	6.98E-17	1.21	4.28E-15					
23218	NBEAL2	1.22	8.29E-17	1.18	9.96E-10	1.06	1.71E-03	1		
4026	LPP	1.16	1.69E-16	1.13	1.16E-09	1.30	4.80E-05	1	0.61	0.51
23053	KIAA0913	1.17	2.72E-16	1.18	3.33E-14	1.20	1.29E-05	0.3	0.61	0.48
10482	NXF1	1.14	3.69E-16	1.16	7.99E-17	1.07	6.24E-04	1		
**Top 10 AD DEGs not MCI DEGs in blood**										
51186	WBP5	0.96	8.95E-10	0.98	1.22E-02	1.12	1.06E-08	2.50E-04		0.49
10287	RGS19	1.11	1.12E-09	1.04	7.18E-02	1.10	3.48E-08	8.19E-04		
9147	SDCCAG1	0.93	2.53E-09	0.97	5.39E-02	1.20	1.24E-05	0.29		
3276	PRMT1	0.92	9.52E-09	0.96	1.24E-02	0.91	6.55E-05	1	−0.48	
51150	SDF4	0.91	1.02E-08	0.97	7.30E-02	1.04	8.70E-01	1		
10623	POLR3C	0.94	1.16E-08	0.97	3.34E-02	0.90	1.53E-05	0.36	−0.49	−0.46
253018	HCG27	1.16	1.71E-08	1.06	6.14E-02	1.07	2.34E-02	1		
4850	CNOT4	0.95	3.47E-08	0.97	2.20E-02	1.05	3.27E-01	1	0.53	0.47
80315	CPEB4	1.14	6.41E-08	1.07	2.17E-02	1.22	5.00E-01	1		
23001	WDFY3	1.08	7.36E-08	1.04	3.49E-02	1.00	1.79E-03	1		0.48
**Top 10 MCI DEGs not AD DEGs in blood**										
587	BCAT2	1.03	3.29E-02	1.09	8.93E-10	1.17	7.92E-10	1.86E-05	0.66	0.53
23338	PHF15	1.03	2.59E-01	1.13	1.61E-09	1.11	1.72E-01	1		
26284	ERAL1	1.05	1.06E-02	1.12	5.20E-09	1.01	1.25E-01	1		
8036	SHOC2	0.96	1.91E-02	0.89	7.53E-09	0.88	1.34E-03	1		
23450	SF3B3	1.05	1.56E-02	1.12	9.88E-09	0.97	7.38E-02	1		
4289	MKLN1	0.95	2.11E-02	0.87	1.18E-08	1.24	2.75E-09	6.48E-05	0.67	0.58
57666	KIAA1545	1.04	1.37E-02	1.09	1.28E-08	1.10	5.90E-02	1	0.51	0.49
9236	CCPG1	0.93	1.06E-02	0.85	2.30E-08	0.94	5.04E-04	1		
94241	TP53INP1	0.94	2.18E-02	0.87	3.73E-08	1.26	2.37E-06	5.58E-02	0.66	0.56
78987	CRELD1	1.03	1.32E-02	1.08	4.00E-08	1.01	3.92E-01	1		

Data shown are from (top rows) the top 10 up-regulated and the top 10 down-regulated DEGs in AD blood that are also DEGs in MCI blood; (middle rows) the top 10 DEGs in AD blood that are not also DEGs in MCI blood; (bottom rows) the top 10 DEGs in MCI blood that are not also DEGs in AD blood. In addition, all these DEGs in blood were mapped to DEGs in the brain PFC region^7^ (columns 7 to 9) and we show their correlation coefficient braak stage and brain frontal atrophy^4^ in patients with AD. FC represents Fold Change in gene expression.

### Validation using Gene expression in other blood datasets

Among the 374 DEGs identified in the GSE6613 validation dataset (see Methods and Supplementary Table 2), 357 were included in the merged discovery dataset. Although DEGs identified in the discovery dataset had an enrichment of DEGs identified in GSE6613 (OR = 2.37, 95%CI 1.91–2.95, pval = 8.35E-15; and OR = 2.78, 95%CI 2.24–3.46, pval = 2.74E-21, for AD and MCI respectively), only three of the top DEGs listed in Table 1 were re-discovered in GSE6613, namely *WDFY3, TCIRG1*, and *NEMF/SDCCAG1*.

In the ADNI2 dataset, we identified 416, 630, and 157 DEGs (unique genes) for AD, early MCI (EMCI) and late MCI (LMCI) disease status respectively (see Supplementary Table 2). Both AD-DEGs and MCI-DEGs identified in the merged discovery cohort were enriched with DEGs identified in ADNI2 AD (OR = 1.88, 95%CI 1.53–2.33, pval = 6.11E-09; OR = 2.02, 95%CI 1.65–2.48, 9.67E-12, for AD and MCI respectively, Supplementary Fig. 4). None of the top DEGs listed in Table 1 were re-discovered in the ADNI2 AD dataset. However, *HELZ* was identified as an early MCI-DEG in the sub-cohort of ADNI2 with APOE4 = 1 genotype. This gene had a 12% up-regulation in both blood of AD and blood of MCI in the merged discovery dataset. An exome sequencing study revealed that variants in *HELZ* are associated with intellectual disability^24^. HELZ functions as a RNA helicases, and RNA helicases are involved in almost every RNA related process, including transcription, splicing, ribosome biogenesis, translation and degradation. Therefore, HELZ may have associations with the pathogenesis of neurodegenerative disease including AD^25^.

### Pathway Analysis shows large overlap between blood and brain

**Figure 1:**
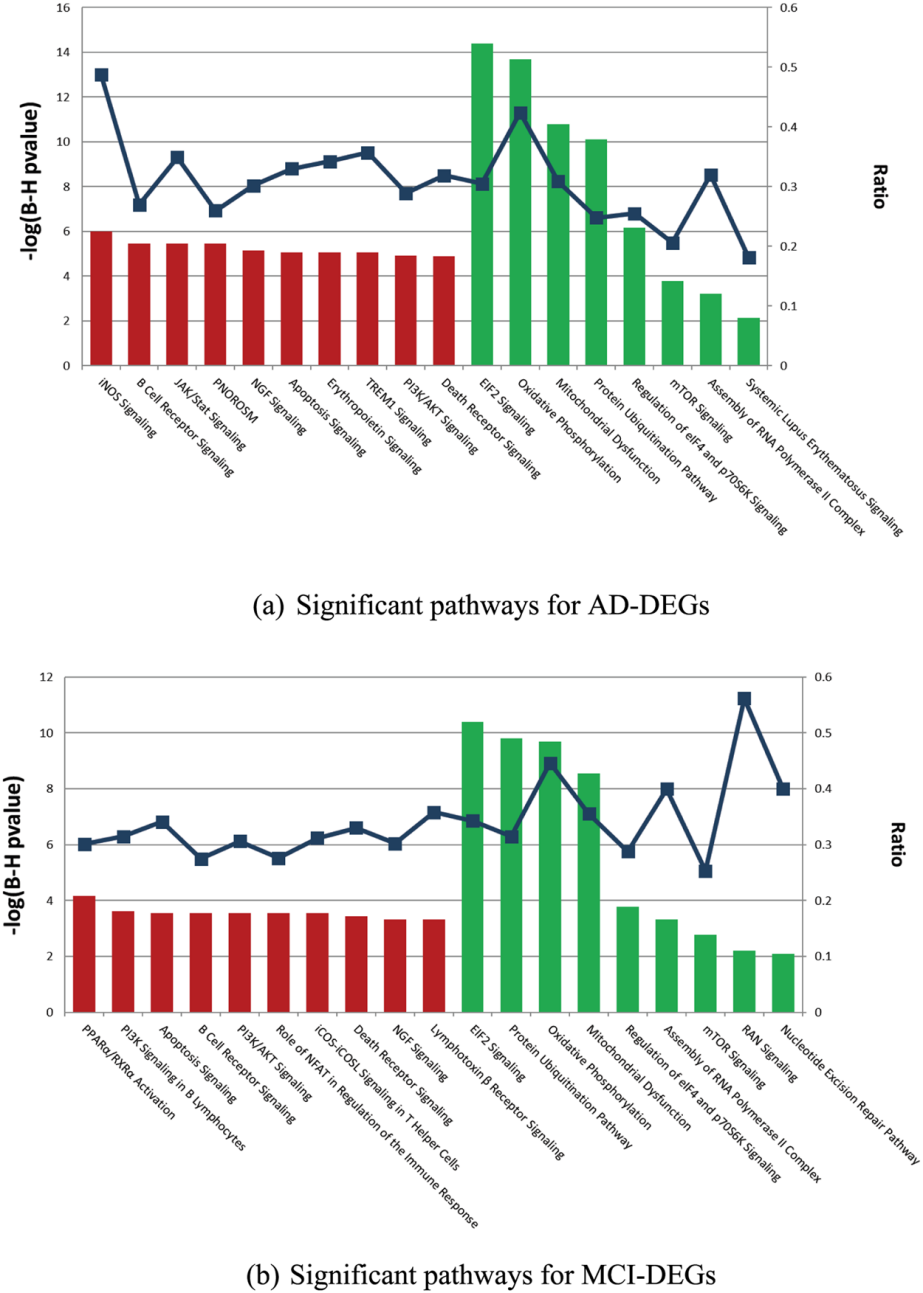
Significant pathways identified by IPA in the blood dataset. IPA was applied to the DEGs identified from the merged blood datasets (GSE63060 and GSE63061). We show the top ten significant pathways identified for the up-regulated DEGs (red bar) and pathways identified for the down-regulated DEGs (green bar). The dark blue curve shows the ratio between the number of DEGs and the total number of genes in each of these pathways (entire list of IPA pathways is in Supplementary Table 3). (**a)** Significant pathways for AD-DEGs. Top ten significant pathways identified for the up-regulated DEGs and eight pathways identified for the down-regulated DEGs in AD. (**b**) Significant pathways for MCI-DEGs. Top ten significant pathways identified for the up-regulated DEGs and nine pathways identified for the down-regulated DEGs in MCI.

For the up-regulated AD-DEGs in blood, 119 significant canonical pathways were identified, including iNOS Signalling (BH.pval = 9.77E-7, ratio = 21/43); B-Cell Receptor Signalling (BH.pval = 3.55E-6, ratio = 48/178); JAK/Stat Signalling (BH.pval = 3.55E-6, ratio = 29/83); and Production of Nitric Oxide and Reactive Oxygen Species in Macrophages (PNOROS, BH.pval = 3.55E-6, ratio = 50/192). For the down-regulated AD-DEGs, only eight significant pathways were identified (Fig. 1 and Supplementary Table 3) including EIF2 Signalling (BH.pval = 3.98E-15, ratio = 64/210); Oxidative Phosphorylation (BH.pval = 2.00E-14, ratio = 39/92); Mitochondrial Dysfunction (BH.pval = 1.58E-11, ratio = 47/152); and Protein Ubiquitination (BH.pval = 7.92E-11, ratio = 63/254), Similarly, we identified 63 and nine significant canonical pathways for up- and down-regulated blood MCI-DEGs respectively. A total of 53 and seven significant pathways were overlapping between AD and MCI for up- and down-regulated DEGs respectively including the top pathways mentioned above. Therefore, 83.3% (60 out of 72) significant pathways identified in MCI were also identified in AD. In our previous gene expression meta-analysis, we identified 168 significant pathways in the brain PFC region^7^, and 60.1% of these (101 out of 168) were identified in either blood AD or blood MCI, including PNOROS (BH.pval = 1.26E-12, ratio = 44/180), NF*k*B Signalling (BH.pval = 1.26E-11, ratio = 41/173), iNOS Signalling (BH.pval = 5.37E-7,ratio = 15/44), Mitochondrial Dysfunction (BH.pval = 2.24E-06, ratio = 37/172), and Oxidative Phosphorylation (BH.pval = 4.27E-4, ratio = 24/110). Some pathways were only identified in either blood AD or blood MCI, but not in brain PFC region with AD, such as EIF2 Signalling, Protein Ubiquitination, and mTOR Signalling (see Supplementary Table 3).

### Differentially expressed genes not uniform across brain regions

**Figure 2:**
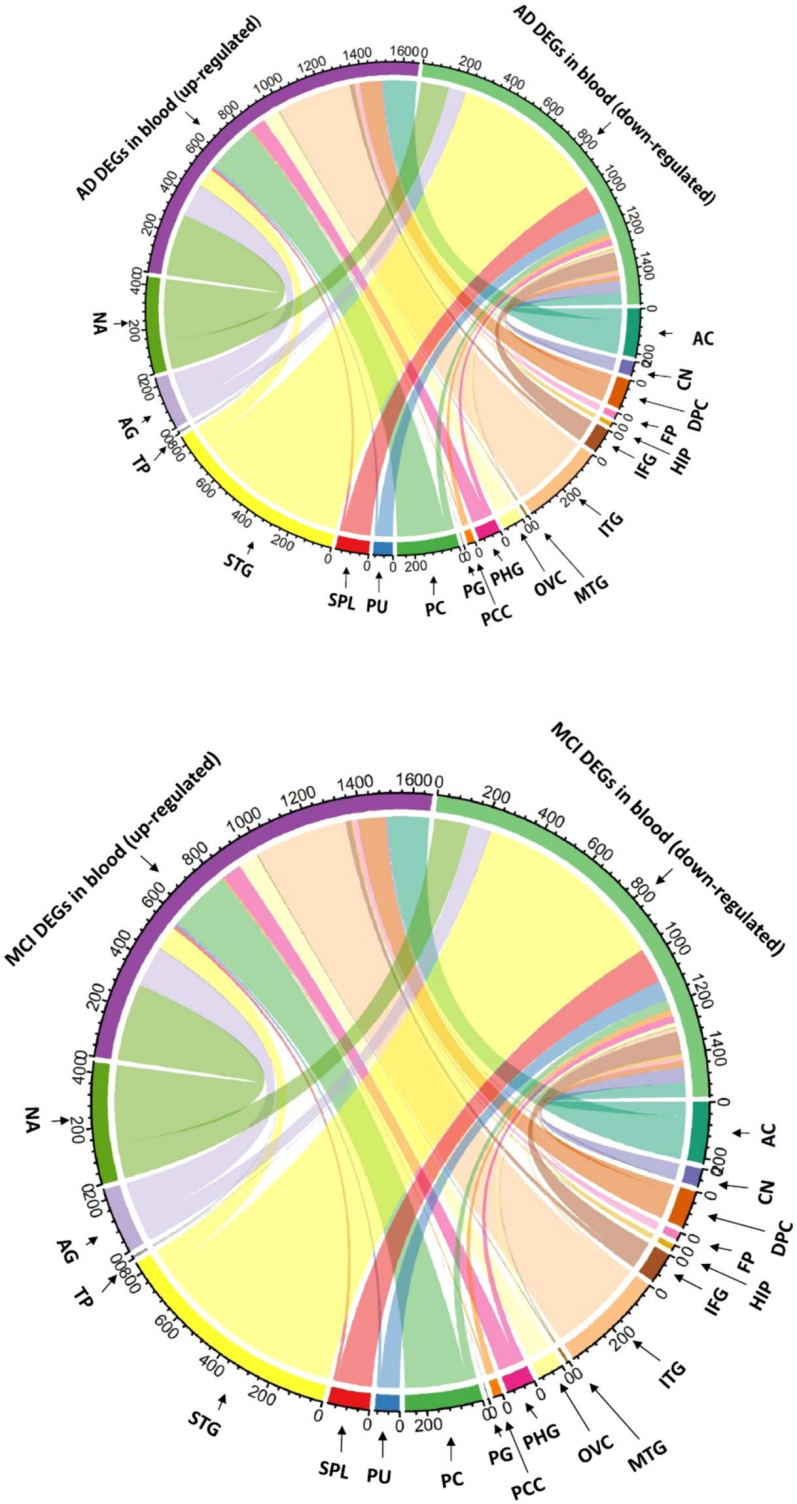
Number of DEGs common to both the blood and the different brain regions. Overlap between DEGs (up-regulated and down-regulated) identified in the merged blood datasets and DEGs identified in each of the 10 brain regions is shown as an arc, the area of which is proportional to the number of overlapping DEGs (see full name of brain region in Table 2).

In total, we identified 5552 AD-DEGs (unique genes) in 19 brain sub-regions (Supplementary Table 4), with the numbers of DEGs varying from 14 (Precentral Gyrus) to 1904 (Superior Temporal Gyrus), and an average of 453 DEGs in each region. With such a divergent distribution across 19 brain regions (Table 2), we did not identify any super genes which were DEGs in all 19 brain regions. Two genes (*AKAP9*, *NEBL*) were identified as DEGs in eight brain regions, and 3640 DEGs were identified from only a single region. 1048 of these DEGs (18.9%) were identified in our previous meta-analysis in brain PFC region (OR = 1.78, 95%CI 1.64–1.94, pval < 3.53E-42). Figure 2 illustrates the DEGs in these 19 brain regions and the overlap with AD-DEGs or MCI-DEGs in blood. Among these 19 brain regions, Prefrontal Cortex (PC), Occipital Visual Cortex (OVC), and Dorsolateral Prefrontal Cortex (DPC) are the top three regions with the highest proportion of brain DEGs mapped to blood. Only 15% of brain DEGs in hippocampus (HIP) were identified as AD-DEGs in blood. In addition, the mappings of brain AD-DEGs to blood AD-DEGs and brain AD-DEGs to blood MCI-DEGs, were highly associated (R = 0.80, pval < 3.33E-05, Pearson test, Table 2).

**Table 2:** Numbers of DEGs identified in brain regions and their overlapping in blood.

Region	Brain AD DEGs			Blood AD DEGs				Blood MCI DEGs				Region names
	Up	Down	All	Up	Down	All	Ratio	Up	Down	All	Ratio	
PFC	526	94	620	182	29	211	0.34	240	38	278	0.45	Prefrontal Cortex
OVC	154	73	227	62	15	77	0.34	77	16	93	0.41	Occipital Visual Cortex
DPC	238	87	325	79	17	96	0.30	109	26	135	0.42	Dorsolateral Prefrontal Cortex
STG	297	1607	1904	65	494	559	0.29	90	749	839	0.44	Superior Temporal Gyrus
AC	391	125	516	109	39	148	0.29	161	57	218	0.42	Anterior Cingulate
ITG	839	104	943	259	9	268	0.28	359	15	374	0.4	Inferior Temporal Gyrus
SPL	29	313	342	9	85	94	0.27	14	136	150	0.44	Superior Parietal Lobule
PU	46	201	247	8	58	66	0.27	12	77	89	0.36	Putamen
PCC	16	63	79	1	20	21	0.27	7	25	32	0.41	Posterior Cingulate Cortex
NA	864	527	1391	221	109	330	0.24	300	137	437	0.31	Nucleus Accumbens
IFG	94	241	335	15	62	77	0.23	22	88	110	0.33	Inferior Frontal Gyrus
CN	52	155	207	5	39	44	0.21	7	58	65	0.31	Caudate Nucleus
PG	5	9	14	1	2	3	0.21	1	1	2	0.14	Precentral Gyrus
AG	559	251	810	101	58	159	0.20	155	82	237	0.29	Amygdala
PHG	254	117	371	54	21	75	0.20	75	28	103	0.28	Parahippocampal Gyrus
TP	50	8	58	7	3	10	0.17	7	3	10	0.17	Temporal Pole
MTG	21	10	31	3	2	5	0.16	6	4	10	0.32	Middle Temporal Gyrus
FP	98	34	132	13	7	20	0.15	18	12	30	0.23	Frontal Pole
HIP	40	32	72	2	9	11	0.15	7	13	20	0.28	Hippocampus

This table shows the number of DEGs identified in 19 brain regions, and their overlap with DEGs in the blood. The Ratio column in the table indicates the proportion of brain DEGs which are also DEGs in blood. For example, there are 620 DEGs identified in the brain Prefrontal Cortex (PFC) region, 211 of them (Ratio = 0.34) are also DEGs in AD blood, and 278 of them (Ratio = 0.45) are DEGs in MCI blood. The PFC region has the highest proportion of DEGs which are also DEGs in the blood, both for AD and MCI patients.

### Gene-based GWAS reveals potential new risk genes

In total, 18229 genes were identified in the IGAP stage 1 GWAS dataset by MAGMA, including all of the 39 GWAS risk genes in AD except *INPP5D*. Sixty seven MAGMA genes passed BF.pval < 0.05, including 17 AD risk genes, and 15 AD-DEGs and 20 MCI-DEGs in blood (Table 3). Among them, *MS4A6A, MS4A4A, ABCA7, HLA-DRA, MTSS1L, NDUFS3*, and *CD2AP* were identified as DEGs in the brain PFC region in our previous brain meta-analysis; Thirteen of them were differentially expressed in at least one brain region. *ABCA7* showed 17%-, 19%-, and 13% significant expression fold changes in blood of AD, blood of MCI and brain of AD respectively; this gene may thus be a potential biomarker for early diagnosis. *MS4A6A* showed >10% down-regulation in blood, and >43% up-regulation in brain; *NDUFS3* was >10% down-regulated, and *HMHA1* >9% up-regulated in blood and brain. Although *HMHA1* is not a risk gene in AD, it has been reported that methylation sites in this gene have a strong relationship to *ABCA7* and AD pathologies^26^. In addition, *BCL3*, a proto-oncogene candidate, might be a potential novel risk gene for AD, because it was 27% up-regulated in AD brain and identified as a DEG in both AD blood and MCI blood. Supplementary Table 5 indicates the 751 IGAP MAGMA genes (nominal pval < 0.01) and the most significant SNPs in their 20kbp up/downstream regions. We identified 281 and 119 genes at 0.05 or 0.01 significance level respectively when FDR testing was applied.

**Table 3:** Results of gene-based GWAS analysis.

Entrez Gene	Name	CHR	#SNPS	BF.pval	Blood AD FC	Blood AD BH.pval	Blood MCI FC	Blood MCI BH.pval	Brain AD FC	Brain AD BF.pval
10452	TOMM40*	19	139	2.72E-150	1.03	0.14	1.06	6.87E-04	0.92	1.00
341	APOC1	19	82	2.70E-135	1.00	0.67	1.01	0.25	1.27	0.85
348	APOE*	19	97	1.52E-131					1.12	1.00
5819	PVRL2	19	268	3.34E-125	0.99	0.52	1.03	0.43	1.05	1.00
4059	BCAM	19	129	1.60E-38	1.00	0.69	1.00	0.73	1.04	1.00
5971	RELB	19	115	1.32E-14	1.08	6.64E-07	1.08	1.79E-05	1.00	1.00
602	BCL3	19	141	4.10E-13	1.07	1.41E-02	1.07	2.25E-02	1.27	6.18E-05
388551	CEACAM16	19	171	1.16E-11					1.02	1.00
346	APOC4	19	102	4.23E-10					1.04	1.00
1209	CLPTM1	19	198	1.39E-09	1.08	8.21E-05	1.15	1.25E-10	0.96	1.00
90332	EXOC3L2	19	148	3.12E-09	1.00	0.80	1.00	0.76	1.00	1.00
344	APOC2	19	105	1.06E-08					1.02	1.00
1191	CLU*	8	148	2.15E-08	1.00	0.96	1.00	0.83	1.31	1.12E-05
79090	TRAPPC6A	19	94	4.14E-08	0.99	0.62	1.01	0.46	0.93	0.02
2041	EPHA1*	7	125	6.05E-08	1.01	0.57	1.03	0.092	1.01	1.00
284353	NKPD1	19	73	9.90E-08					0.97	1.00
643680	MS4A4E	11	206	9.86E-07						
8301	PICALM*	11	396	1.06E-06	1.01	0.55	0.93	1.90E-02	0.95	1.00
11129	CLASRP	19	111	1.27E-06	1.01	0.32	1.00	0.79	1.18	1.00
388552	BLOC1S3	19	70	1.49E-06					0.98	1.00
284352	PPP1R37	19	216	1.95E-06					0.97	1.00
64231	MS4A6A*	11	102	2.15E-06	0.89	4.16E-04	0.83	5.25E-07	1.43	0.00
5817	PVR	19	125	3.92E-06	1.00	0.78	1.01	0.20	0.96	1.00
2206	MS4A2	11	131	5.56E-06	0.99	0.48	0.98	0.22	0.98	1.00
51338	MS4A4A*	11	201	6.82E-06	0.98	1.08E-02	0.97	1.14E-05	1.44	3.38E-07
10347	ABCA7*	19	234	8.97E-06	1.17	7.97E-10	1.19	2.68E-09	1.13	2.47E-03
1378	CR1*	1	373	2.01E-05	1.08	9.70E-05	1.05	1.39E-02	1.03	1.00
23624	CBLC	19	118	2.83E-05					1.00	1.00
274	BIN1*	2	348	2.95E-05	1.04	0.11	1.11	1.81E-04	0.96	1.00
147710	IGSF23	19	155	3.08E-05					0.99	1.00
338398	TAS2R60	7	76	6.74E-05					1.00	1.00
79760	GEMIN7	19	138	7.40E-05	0.99	0.21	1.00	0.53	0.87	1.00
1839	HBEGF	5	77	9.99E-05	1.00	0.99	1.00	0.66	1.07	1.00
7791	ZYX	7	100	2.52E-04	1.13	1.51E-05	1.18	2.42E-07	1.03	1.00
23526	HMHA1	19	221	2.92E-04	1.09	3.77E-06	1.14	7.87E-10	1.20	4.05E-07
56971	CEACAM19	19	117	3.03E-04	1.01	0.14	1.00	0.56	1.07	1.00
1379	CR1L	1	420	4.27E-04					1.04	1.00
162979	ZNF296	19	98	4.85E-04	1.04	4.03E-02	1.10	2.34E-07	1.00	1.00
23403	FBXO46	19	82	9.57E-04	1.05	5.69E-06	1.08	5.47E-07	1.05	1.00
6653	SORL1*	11	354	1.25E-03	1.21	2.80E-08	1.31	1.02E-12	0.89	1.00
6688	SPI1	11	127	2.33E-03	1.13	3.72E-05	1.13	5.42E-04	1.09	1.00
3122	HLA-DRA	6	722	2.46E-03	0.95	0.076	0.90	1.43E-03	1.40	9.51E-08
3123	HLA-DRB1*	6	1550	2.83E-03	0.93	0.68	1.08	0.65	1.41	2.25E-03
1265	CNN2	19	203	2.88E-03	1.03	0.50	1.06	0.08	1.23	2.69E-03
245802	MS4A6E	11	200	3.10E-03					1.05	1.00
1135	CHRNA2	8	191	3.61E-03	1.03	0.21	1.06	3.74E-03	1.00	1.00
92154	MTSS1L	16	172	3.74E-03					1.24	1.16E-02
399888	FAM180B	11	35	5.27E-03	1.00	0.52	1.00	0.73	1.03	1.00
114971	PTPMT1	11	43	5.58E-03					0.92	1.00
4722	NDUFS3*	11	41	7.22E-03	0.90	7.38E-10	0.90	1.78E-08	0.86	1.32E-02
388553	BHMG1	19	76	7.26E-03					1.03	1.00
55709	KBTBD4	11	37	7.75E-03	1.02	0.087	1.02	3.74E-02	0.88	3.63E-02
945	CD33*	19	119	7.75E-03	1.04	0.058	1.03	0.17	1.22	9.72E-07
1762	DMWD	19	89	9.46E-03	1.05	1.56E-04	1.06	1.40E-06	0.96	1.00
2185	PTK2B*	8	486	1.00E-02	1.06	2.19E-03	1.03	0.15	0.99	1.00
23788	MTCH2*	11	71	1.02E-02	1.00	0.66	1.00	0.71	0.86	1.00
55697	VAC14	16	274	1.57E-02	1.04	8.06E-05	1.06	1.76E-08	0.99	1.00
1760	DMPK	19	73	2.21E-02	1.00	0.72	1.01	2.60E-02	1.09	1.00
23607	CD2AP*	6	451	2.37E-02	0.99	0.12	0.97	1.53E-03	1.15	4.08E-07
3117	HLA-DQA1	6	2022	2.42E-02	0.92	0.14	0.98	0.74	1.20	1.00
932	MS4A3	11	142	2.46E-02	0.91	1.42E-03	0.88	1.58E-04	0.96	1.00
147912	SIX5	19	60	2.48E-02	1.00	0.84	1.01	0.19	1.27	1.92E-04
114900	C1QTNF4	11	38	2.83E-02					0.82	1.89E-03
23360	FNBP4	11	140	3.08E-02	0.98	0.58	0.96	0.19	1.00	1.00
56244	BTNL2	6	613	3.48E-02					1.01	1.00
28955	DEXI	16	138	4.19E-02	1.00	0.88	1.02	0.18	1.03	1.00
79841	AGBL2	11	128	4.83E-02					1.00	1.00

This table lists 67 genes identified by MAGMA (BF.pval < 0.05) from the IGAP stage 1 GWAS dataset, and compares their expression (fold-change and p-value) in AD and MCI blood datasets, and in the brain dataset from our previous study^7^. AD GWAS risk genes are marked with an asterisk, “*”. The chromosome and the number of SNPs for each of these genes within 20 kbp up- and downstream regions are shown in the third and fourth columns. BF.pval indicates Bonferroni corrected p-value, while BH.pval indicates Benjamini & Hochberg corrected p-value.

DEGs in blood did not show any enrichment for these IGAP MAGMA genes at the stringent significance level (BF.pval > 0.05). However, if we apply nominal pval < 0.01 for MAGMA (751 genes identified), both AD-DEGs and MCI-DEGs in blood show enrichment in IGAP genes (OR = 1.33, 95%CI 1.11–1.61, pval = 2.45E-03; OR = 1.36, 95%CI 1.14–1.62, pval = 5.33E-04, respectively). We previously identified 3124 AD-DEGs in the brain PFC region^7^, and those DEGs had enriched MAGMA genes either for BF.pval < 0.05 or nominal pval < 0.01 (OR = 2.27, 95%CI 1.23–4.02, pval = 5.67E-03; OR = 1.23, 95%CI 1.00–1.51, pval = 4.64E-02 respectively). These results revealed the significant associations between genomics and gene expression in AD.

### Creation of potential biomarker panels by machine learning

Our aim here was to identify a set of biomarkers and classification models (classifiers) which can discriminate patients with AD from healthy control subjects, e.g. 143 patients with AD from 104 controls in GSE63060 or 102 patients with AD from 78 controls in GSE63061. We trained classifiers in one dataset and tested them in the other dataset (see Methods).

**Figure 3:**
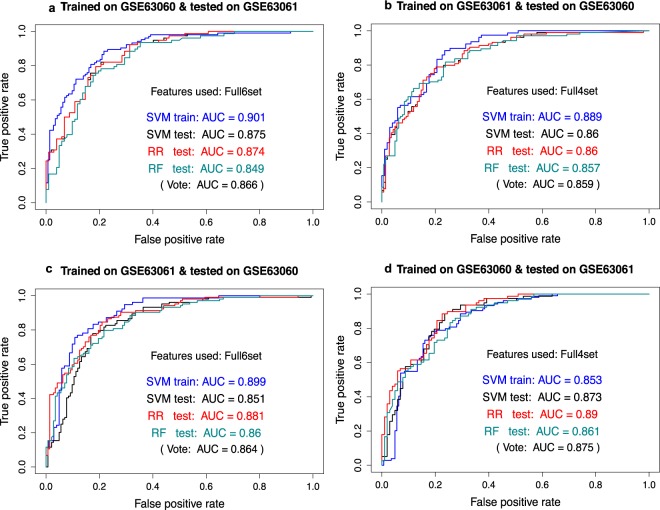
Classification performance of biomarker panels. Different machine learning models were trained in one blood dataset (GSE63060 or GSE63061) and tested in the other (GSE63061 or GSE63060). Results shown from the different ML models in (**a**,**c**) all use the same panel of six features (panel Full6set), while ML models in (**b**,**d**) use one with four features (panel Full4set). Full6set contains six probesets, i.e. ILMN_2097421 (MRPL51), ILMN_2189933 (RPL36AL), ILMN_1695645 (CETN2), ILMN_1703617 (AHSA1), ILMN_2237746 (ING3), and ILMN_1939297 (GALNT4). Full4set contains four probesets: ILMN_1784286 (NDUFA1), ILMN_2097421 (MRPL51), ILMN_2189933 (RPL36AL) and ILMN_2189936 (RPL36AL). The AUC of vote is the average testing AUCs of SVM, RR, and RF models. See Supplementary Table 6 for detailed performance.

Figure 3a illustrates an optimal six-feature panel (named Full6set) that was identified by measuring area under the curve (AUC) performance for SVM, RR and RF (0.875, 0.874, 0.849 respectively). The voted AUC (the average of the three AUCs) was 0.866 with 0.783 (95%CI: 0.716–0.841) accuracy for voting outcome. The Full6set contains six probesets: ILMN_2097421 (*MRPL51*), ILMN_2189933 (*RPL36AL*), ILMN_1695645 (*CETN2*), ILMN_1703617 (*AHSA1*), ILMN_2237746 (*ING3*), and ILMN_1939297 (*GALNT4*). In Fig. 3b, an optimal four-feature panel (named Full4set) was identified containing ILMN_1784286 (*NDUFA1*), ILMN_2097421 (*MRPL51*), ILMN_2189933 (*RPL36AL*) and ILMN_2189936 (*RPL36AL*). SVM, RR and RF classification models had similar testing AUC performance (0.86, 0.86, 0.857) and accuracy (0.773, 0.765, 0.785) respectively. The voting strategy yielded the average AUC of 0.859 and accuracy of 0.781 (95%CI: 0.725–0.831) with balanced sensitivity (0.776) and specificity (0.788). See Supplementary Table 6 for further details.

All features in Full6set and Full4set were down-regulated DEGs in the blood merged discovery dataset, except *GALNT4* which was an up-regulated DEG (Supplementary Table 2); the two common features, ILMN_2097421 (*MRPL51*) and ILMN_2189933 (*RPL36AL*), were the top DEGs in the blood but not in the brain. In order to test the robustness of the classification models and features used, we swapped the training dataset and testing dataset, i.e. we trained classification models in GSE63060 using Full4set then tested in GSE63061, and we trained models in GSE63061 using Full6set and tested in GSE63060. Their testing performances are illustrated in Fig. 3c,d, and Supplementary Table 6. The robustness of the selected features was also tested by random selection (Supplementary Fig. 6). The models using Full6set demonstrated similar classification performances to the models using Full4set. Voting AUC for Full6set models were 0.866 and 0.864 in the two testing datasets (GSE63060 and GSE63061 respectively) with an average of 0.865. For Full4set models, the values were 0.859, 0.875 with an average of 0.867. Moreover, when we used the models trained from AD vs. controls to discriminate MCI from controls, most of the MCI (>72%) were predicted to be AD (Supplementary Table 7). Supplementary Fig. 7 shows the boxplots and swarm plots of each of the features in Full4set where MCI samples were also included, which demonstrates that each of the features had good classification performance.

## Discussion

In this study, we observed that in blood samples more DEGs were identified comparing MCI to controls than comparing AD to controls. This suggests that the trajectory from control to MCI to AD is surely not linear. In addition, under the current classification of MCI there are many clinical entities, not all evolving to AD in the same way or time (some MCI even revert to control). Therefore, it is possible that the increased differences we observed between MCI and controls reflect the MCI’s dynamic and heterogeneous state. On the contrary, overt AD is a more stable clinical entity with possibly a more defined gene expression signature. We also observed that AD-DEGs tended to have the same regulation direction as the MCI-DEGs in blood (only a few genes were identified as DEGs comparing AD to MCI samples), and the majority of those AD-DEGs that overlapped in the blood and brain showed consistent directions of regulation, suggesting the biomarkers to be investigated in blood can be potential early diagnostic signatures. Our study shows evidence for a role of ribosomal dysfunction. In blood, the top 10 up- and down-regulated AD-DEGs were also identified as MCI-DEGs, and included ribosomal protein genes such as *MRPL51*, *RPL36AL*, and *RPS25*. Ribosome dysfunction is an early event in AD^27^, and the abnormal tau-ribosomal interactions in tauopathy lead to a decrease in RNA translation^28^. Two recent studies reported that reducing ribosomal protein S6 kinase 1 expression improves spatial memory and synaptic plasticity in a mouse model of AD^29^, and there are striking overlaps between non-steroidal anti-inflammatory (NSAID) drugs-induced changes and gene expression in the blood of AD patients in the ribosome and oxidative phosphorylation pathways^30^. A novel mutation discovered in the gene *NDUFA1* may also lead to a progressive mitochondrial complex I specific neurodegenerative disease^31^. *TYK2* and *STAT3* were identified as up-regulated DEGs in both blood and brain (Supplementary Table 2). Tyk2/Stat3 signalling mediates beta-amyloid-induced neuronal cell death in AD^32^. *TYK2* encodes a member of the tyrosine kinase specifically for the Janus kinases (JAKs) protein families, and inhibition of JAK1/JAK3 may provide an efficient therapeutic agent for the treatment of inflammatory diseases^33^ which might benefit AD patients as well since inflammation drives progression of AD^34^. It is interesting to note that *TCIRG1* showed a greater than 20% up-regulation in blood of AD, blood of MCI and brain of AD. Mutations in this gene can cause lower absolute neutrophil count and may be responsible for infantile malignant osteopetrosis (IMO) disease^35,36^. However, its role in AD or dementia is not yet proven, and it may be related to neutrophil function and immunity.

We observed that DEGs in blood have a high potential to be identified as DEGs in brain prefrontal cortex region (PFC) through enrichment analysis. Table 2 shows that DEGs in brain PFC, Superior Temporal Gyrus (STG), Inferior Temporal Gyrus (ITG) regions are commonly DEGs in blood. Few DEGs were identified in brain hippocampus (HIP) region due to the large shrinkage in HIP that radically reduces gene expressions, and these DEGs have a low likelihood of being identified as DEGs in blood. It is well known that the hippocampus, a critical region for learning and memory, is especially vulnerable to damage at early stages of AD, hippocampal volume is one of the best AD biomarkers for diagnosis. The brain temporal cortex including STG, ITG, HIP, etc. plays a critical role in cognitive processes, language comprehension, memory formation and recall^6^. Functional segmentation analysis revealed that AD patients exhibit stronger hippocampus-PFC functional connectivity^37^. Actually 27.8% of all the DEGs in brain (1544/5552) are also DEGs in AD blood with a significant enrichment (OR = 1.27, 95%CI: 1.18–1.38, pval = 9.8e-10, Fisher test); 2154 DEGs in brain are also DEGs in MCI blood with an enrichment (OR = 1.44, 95%CI 1.34–1.55, pval = 2.2e-16, Fisher test). This shows that gene expression in the blood is a strong representation of gene expressions in the brain.

It has been revealed that mitochondrial dysfunction and oxidative phosphorylation were identified in AD/MCI blood, AD brain and ageing brain, showing the relevance of mitochondrial function in AD^38^. In our present study, we also found strong evidence for dysregulation of the mitochondrial and oxidative phosphorylation pathways in the blood of patients with AD and MCI.

IGAP provides a powerful data resource for the study of AD and it has been explored by several research teams^39,40^. To our knowledge, our study is the first to integrate IGAP with datasets from the blood of AD, blood of MCI and brain of AD. Moreover, recent trans-ethnic GWAS identified five novel AD risk genes^41^ and three of them (*TPBG*, *PFND1*/*HBEGF*, *BZRAP1*-*AS1*) were MAGMA genes in our study. Fourteen out of 39 previously identified risk genes of AD were identified as DEGs in at least one brain region of this disease, including *MAPT*, *APP*, *PSEN1* and *ABCA7*. Genes simultaneously differentially expressed in several brain regions may be AD-relevant risk genes. For example, *AKAP9* was identified as a DEG in eight brain regions including the hippocampus, and two rare mutations in this gene were recently discovered as AD-associated loci by whole exome sequencing^42^.This gene is also at the significance border in blood (BH.pval = 0.033 and 0.012 for AD and MCI respectively). Moreover, Low *et al*. discovered that variants of *NEBL* are relevant to atrial fibrillation (AF) susceptibility^43^, and *NEBL* was identified as a DEG in eight brain regions with AF recognized as a risk factor for cognitive decline and dementia^44^.

Discovering biomarkers in blood for the diagnosis of AD at the earliest and mildest stages is always clinically required and would be hugely beneficial. Recently, Nakamura and colleagues demonstrated the ability of amyloid-β precursor protein APP_669–711_/Aβ_1–42_ and Aβ_1–40_/Aβ_1–42_ ratios, and their composites in plasma to predict brain amyloid-β burden with very high performances^45^. Despite the relatively expensive IP-MS measurement method used, their results bring new hope for blood biomarker-based early diagnosis for AD.

In this study, we identified an optimal classification panel of four features, Full4set, by the LASSO feature selection approach. By applying classifiers with Full4set, 75.4% and 72.7% of MCI were predicted as AD in GSE63061 and GSE63060 respectively (Supplementary Table 7). All features in Full4set were DEGs in blood, and this small feature size panel may have the potential to be applied in Point-of-Care (PoC) diagnostic devices that will be developed and validated in the future.

Our study has a number of limitations. For the two blood datasets (GSE63060 and GSE63061), which are the main focus of this study, we applied multiple testing for DEGs identification. However, for the two validation blood datasets and the brain multiple regions dataset, no DEGs could pass the multiple-testing (BH.pval > 0.05), i.e. no significant genes were identified after allowing for multiple testing. We therefore were forced to apply nominal p-value with a more stringent significance level (<0.01) for DEG detection. The sample sizes used in previous transcriptomic and proteomic studies of AD were generally small, particularly in post-mortem brain studies. Therefore, there was a limited power to identify dysfunctional genes. We observed that most of our DEGs had small effect size, and the small sample sizes (particularly in the brain studies) gave us low statistical powers which resulted in a high level of false positives for DEG detection when nominal p-values were applied. Applying multiple testing may lose information, and alternative network-based approaches could be applied for biomarker discovery^4,46^. In addition, more accurate and sensitive techniques are required to measure such gene expressions, for instance, droplet digital polymerase chain reaction (ddPCR)^47^ and RNA-seq^48^. Aside from sample size, another limitation is that the classification effect of any genetic risk factors was not taken into account due to lack of information availability, e.g. for APOE which may be the most important genetic risk factors for AD^49^. This may be a major limitation as the presence of the *APOE4* allele has been shown to influence the classification algorithms based on medical imaging and cerebrospinal fluid (CSF) biomarkers^50^ (and by our unpublished works). Moreover, our classification model only included gene transcript information and the effect from ageing and gender was adjusted during the data pre-processing. Finally, although AUC-ROC together with Sensitivity/Specificity are frequently used as performance measurements in biomedical research, for example recently in Nakamura and colleagues’ study^45^, it has been reported that Precision/Recall and Area Under Precision Recall (AUPR) can provide more information in imbalanced dataset^51^. We had applied ROC with class-weight adjustment in our model training process, and so we compared these results to those obtained using AUPR to assess the effect of data imbalance (please see Supplementary Fig. 8 and Table 6). In general, AUPR values are a bit lower than AUC-ROC values indicating the effect of data imbalance in our case, and there might have be rooms to improve classification performance by applying AUPR in the feature selection process.

In conclusion, our study revealed that genes differentially expressed in the blood were likely to be differentially expressed in the brain and with the same regulation direction. Common pathways were identified and found to be shared among brain AD, blood AD and ageing brain. We also identified a four-feature panel classification model that discriminated between AD patients and controls with promising performances. A larger cohort study is now necessary to validate the reproducibility of this model’s results perhaps using target-based transcriptional measurement.

## Electronic supplementary material

Supplementary Information

Supplementary Table 2

Supplementary Table 3

Supplementary Table 4

Supplementary Table 5

## Data Availability

This link provides seven datasets: Two initial datasets downloaded from GEO (GSE63060_series_matirx.txt, GSE63061_series_matrix.txt); one merged dataset for DEGs analysis (gse63060_61.merged.exp); two central-scaled datasets for training and testing ML models (files contain 22756 features and disease status for each sample: gse63060_ADMCICtr_Residual_normT_lab.txt, gse63061_ADMCICtr_Residual_normT_lab.txt); and two information files (Samples_gse63060.info, Samples_gse63061.info) extracted from the two GEO datasets. https://figshare.com/s/78839db30d17d3f75aca.
