# Supplementary material for: Systematic Analysis and Biomarker Study for Alzheimer’s Disease
Source: Sci Rep. 2018 Nov 26;8:17394. doi: 10.1038/s41598-018-35789-3 (PMC6255913; doi:10.1038/s41598-018-35789-3)
Supplement: Supplementary file 1 — Supplementary Information [file 41598_2018_35789_MOESM1_ESM.doc]

**Systematic Analysis and Biomarker Study for Alzheimer’s Disease**

Xinzhong Li^1^, Haiyan Wang^2^, Jintao Long^1^, Genhua Pan^3^, Taigang He^4^, Oleg Anichtchik^1^, Robert Belshaw^1^, Diego Albani^5^, Paul Edison^6^, Elaine K Green^1^, James Scott^6^, the Alzheimer’s Disease Neuroimaging Initiative*

^1^Plymouth University Peninsula Schools of Medicine and Dentistry, Drake Circus, Plymouth, PL4 8AA, UK

^2^ Department of Methodology, London School of Economics and Political Science, Houghton St, London WC2A 2AE, UK

^3^School of Computing Electronics and Mathematics, Plymouth University, Drake Circus, Plymouth, PL4 8AA, UK

^4^Molecular and Clinical Sciences Research Institute, St George's, University of London, Cranmer Terrace, London SW17 0RE, UK

^5^Department of Neuroscience, IRCCS - Istituto di Ricerche Farmacologiche "Mario Negri" Via La Masa 19, 20156 Milan – Italy

^6^Department of Medicine, Imperial College London, Du Cane Road, London W12 0NN, UK

Correspondence to: Xinzhong Li ([xinzhong.li@plymouth.ac.uk](mailto:xinzhong.li@plymouth.ac.uk))

Room F122, Darriford Research Facility, Research Way, Plymouth Science Park, Plymouth, Devon, PL6 8BU, UK

*Data used in preparation of this article were obtained from the Alzheimer’s Disease Neuroimaging Initiative (ADNI) database (adni.loni.usc.edu). As such, the investigators

within the ADNI contributed to the design and implementation of ADNI and/or provided data but did not participate in analysis or writing of this report. A complete listing of ADNI investigators can be found at: <http://adni.loni.usc.edu/wp-content/uploads/how_to_apply/ADNI_Acknowledgement_List.pdf>

Supplementary Information content lists:

**Supplementary Figure 1. Overlapping of DEGs in two blood datasets and their merged dataset**.

**Supplementary Figure 2. DEG analysis in the blood discovery dataset.**

**Supplementary Figure 3. DEGs in blood correlated to DEGs in both brain PFC region and in brain pathology.**

**Supplementary Figure 4. DEGs in blood correlated to DEGs in the ADNI2 dataset.**

**Supplementary Figure 5. Comparison of pairwise scatter plots for six-feature model and four-feature model in normalized blood datasets.**

**Supplementary Figure 6**. **Histograms of AUC for randomly selected features in RF models.**

**Supplementary Figure 7. The boxplot and swarm plot of the four-feature model in GSE63060 and GSE63061 datasets when MCI samples were included.**

**Supplementary Figure 8. Precision-recall AUC for each case.**

**Supplementary Table 1.** **Numbers of samples in different datasets used in this study.**

**Supplementary Table 2. Excel file lists all DEGs lists in blood (AD and MCI).**

**Supplementary Table 3. Excel file lists IPA significant pathways for DEGs in blood and overlaps in brain.**

**Supplementary Table 4. Excel file lists DEGs identified in 19 brain regions.**

**Supplementary Table 5. Excel file lists IGAP MAGMA genes and their overlapped DEGs in blood and in brain**.

**Supplementary Table 6.** **Detailed Classification performance of models with four or six features.**

**Supplementary Table 7. Prediction of MCI by using the trained models in AD vs. Control dataset.**

**Supplementary Figure 1. Overlapping of DEGs in two blood datasets and their merged dataset** Overall, 92.9%, (676+1690+1211)/(60+676+1690+1211+213), of AD DEGs in either GSE63060 or GSE63061 are also DEGs in the merged dataset (discovery dataset); and 93.2%, (2349+1848+499)/(241+2349+1848+499+99), of MCI DEGs in these two datasets are also DEGs in the merged one. Those overlapped DEGs have the same regulatory direction, except for 23 out of the 676 AD DEGs between GSE63060 and the Merged set which were in different regulatory directions (i.e. 23/(676+1690) < 1%). In this analysis DEGs refer to probesets with BH.pval<0.01.

**
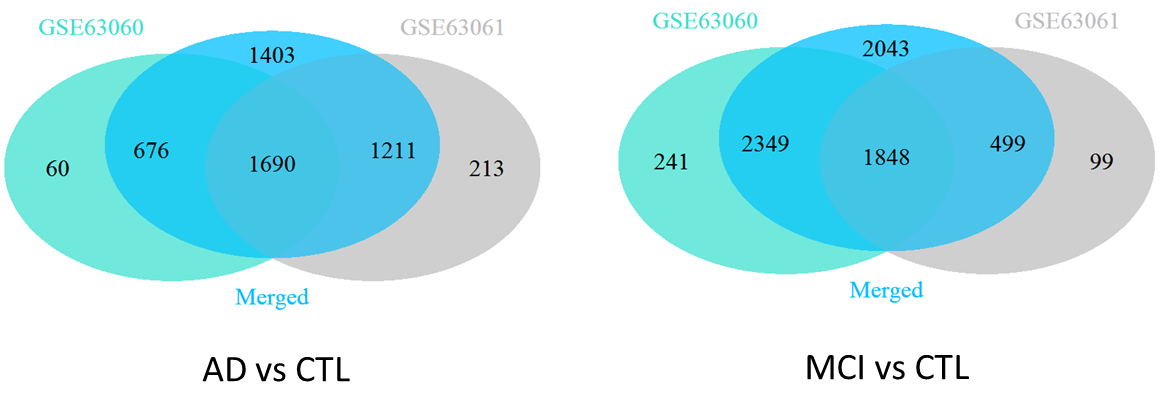
**

**Supplementary Figure 2. DEG analysis in blood discovery dataset.** AD DEGs and MCI DEGs identified in the merged discovery dataset. A total of 4,980 AD DEGs and 6,739 MCI DEGs were identified, and only 82 probesets were identified as differentially expressed between AD and MCI. In the right figure, Red dots are 4,158 overlapped DEGs; light blue dots are 822 DEGs in AD only; blue dots are 2,581 DEGs in MCI only. Overlapped DEGs have the same regulatory direction in AD and MCI. In this analysis DEGs refer to probesets with BH.pval<0.01.

**Supplementary Figure 3. DEGs in blood correlated to DEGs in both brain PFC region and in brain pathology.** The enrichment tests for different cases are:

1. Braak stage: AD: OR=1.42, 95%CI 1.29-1.55, pval=8.62E-14;

MCI: OR=1.48, 95%CI 1.36-1.61, pval=1.74E-19

1. Brain atrophy: AD: OR=1.27, 95%CI 1.14-1.41, pval=8.49E-06;

MCI: OR=1.36, 95%CI 1.23-1.50, pval=6.35E-10

1. DEGs in Brain PFC, 2471 out of 3124 DEGs in brain PFC were included in 16928 blood gene pool

AD: OR=1.48, 95%CI 1.34-1.62, pval=6.28E-16;

MCI: OR=1.39, 95%CI 1.27-1.51, pval=4.90E-13

Sign tests:

AD: 615 out of 789 DEGs common to blood and brain FPFC had same regulatory direction, sign test pval < 2.2e-16

MCI: 877 of 998 common MCI DEGs in blood and AD DEGs were in brain, sign test pval < 2.2e-16

1. DEGs in Ageing: 1,479 ageing genes are included in the 16,928 blood genes pool:

| AD: OR=2.08, 95%CI 1.86-2.33, pval=1.00E-36; MCI: OR=2.00, 95%CI 1.80-2.23, pval=2.93E-36 |
| --- |
|  |

**
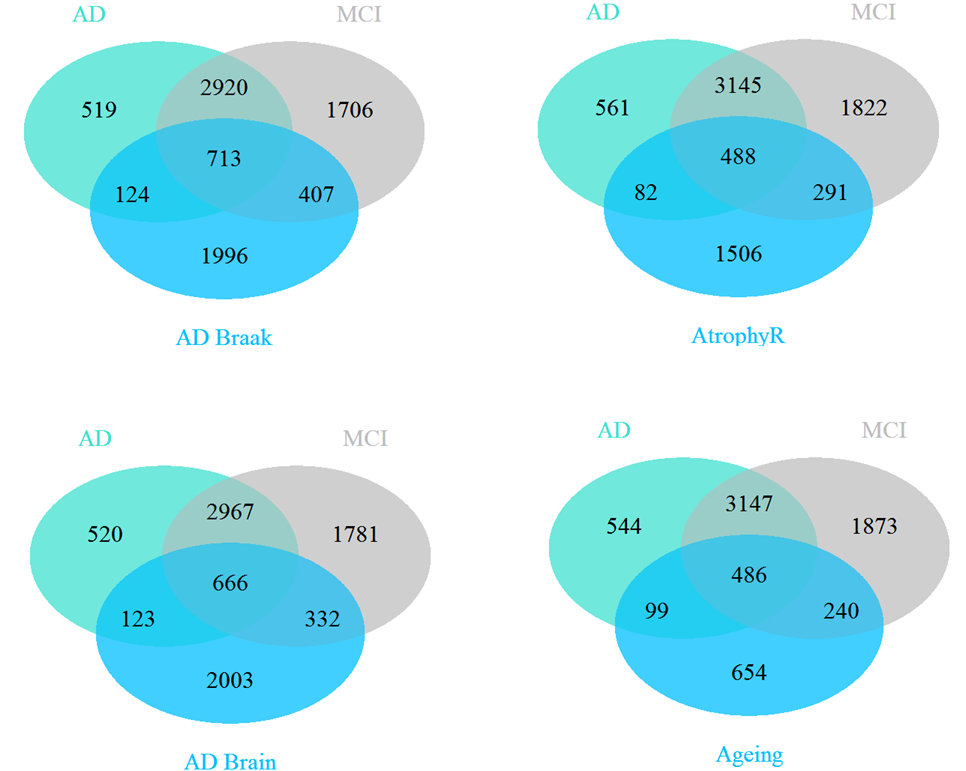
**

**Supplementary Figure 4. DEGs in blood correlated to DEGs in the ADNI2 dataset.** ADNI2 refers to the sub-cohort of original ADNI2 with APOE4=1. AD and MCI refers to the DEGs identified in the discovery dataset. Enrichment analysis results:

1. ADNI2-AD: AD: OR=1.88, 95%CI 1.52-2.33, pval= 6.11E-09; MCI: OR=2.02, 95%CI 1.65-2.48, pval= 9.67E-12
2. ADNI2-EMCI: AD: OR=1.13, 95%CI 0.93-1.37, pval= 0.20; MCI: OR=1.88, 95%CI 1.58-2.23, pval=3.92E-13
3. ADNI2-LMCI: AD: OR=1.79, 95%CI 1.25-2.55, pval= 1.22E-03; MCI: OR=1.82, 95%CI 1.29-2.58, pval= 4.62E-04

**
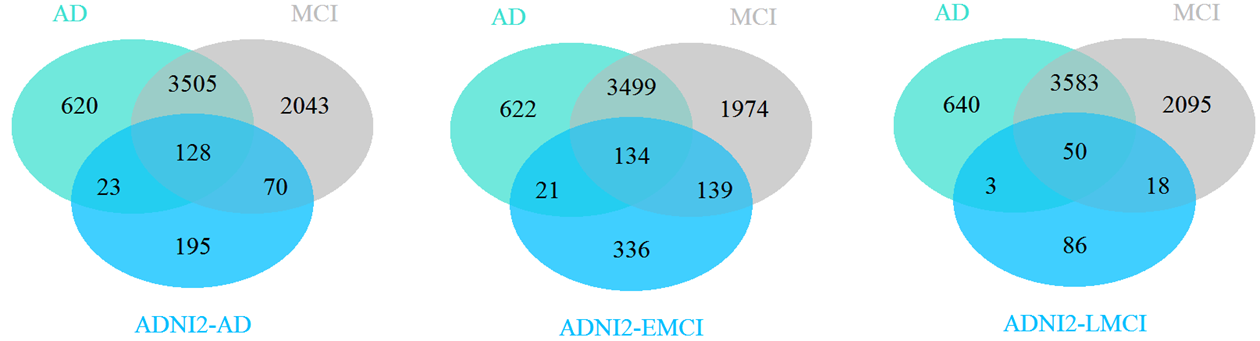
**

**Supplementary Figure 5. Comparison of pairwise scatter plots for six-feature model and four-feature model in the normalized blood datasets.** Full4set model has better linear correlations compared with Full6set model in both GSE63061 and GSE63060 datasets.

**
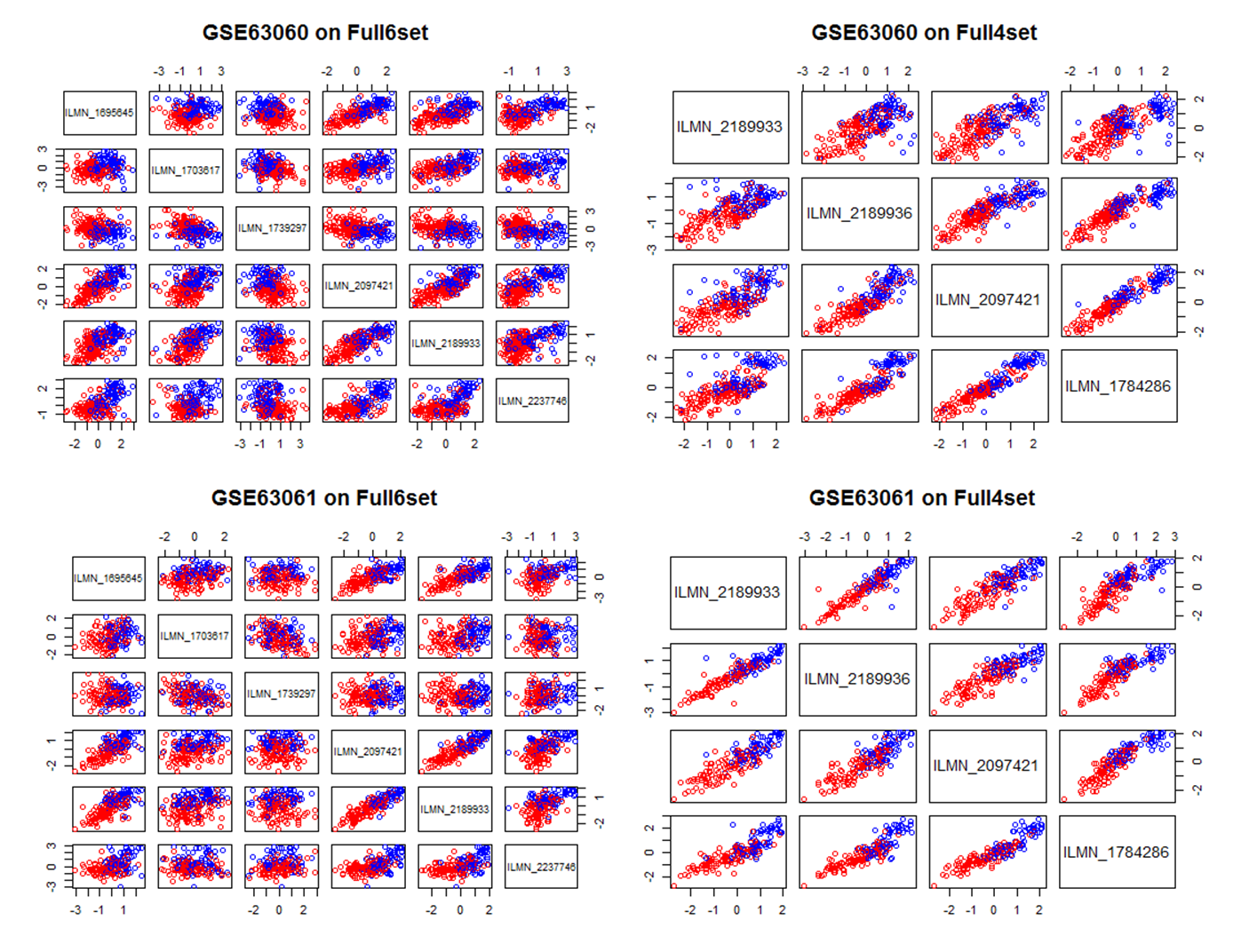
**

**Supplementary Figure 6**. **Histograms of AUC for randomly selected features in RF models**: We repeated 1000 times to train RF models in GSE63060 and test in GSE63061 (left figure), or train in GSE63061 and test in GSE63060 (right figure). Red lines indicate 0.847/0.856 of AUC for 6-feature and 4-feature RF models respectively. In order to test the significance of Full4set and Full6set, we randomly selected six or four features from the full feature pool (22,756 features) and trained the RF models in GSE63060 or GSE63061 for permutation testing. We repeated this process for 1,000 times to get the distribution of the AUC and compared it to the AUC we obtained in Figure 3. No RF model with randomly selected features performed better than our selected models, indicating that Full4set and Full6set were not selected by chance. Without losing generation, we only conducted such permutation testing using the RF model, but believe that the same conclusion would be reached by using SVM and RR models as well.


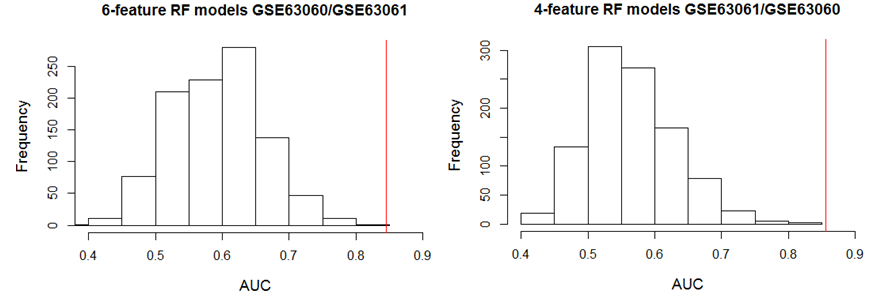


**Supplementary Figure 7. The boxplot and swarm plot of the four-feature model in GSE63060 and GSE63061 datasets when MCI samples were included**. The distribution of MCI was more similar to that of AD than to the Control (CTL), and features in Full4set were down-regulated in MCI. For *NDUFA1*, a subset of Control samples was clearly distinguished from AD samples. There are two feature IDs regarded to gene *RPL36AL*, i.e. ILMN_2189933 and ILMN_2189936.

**
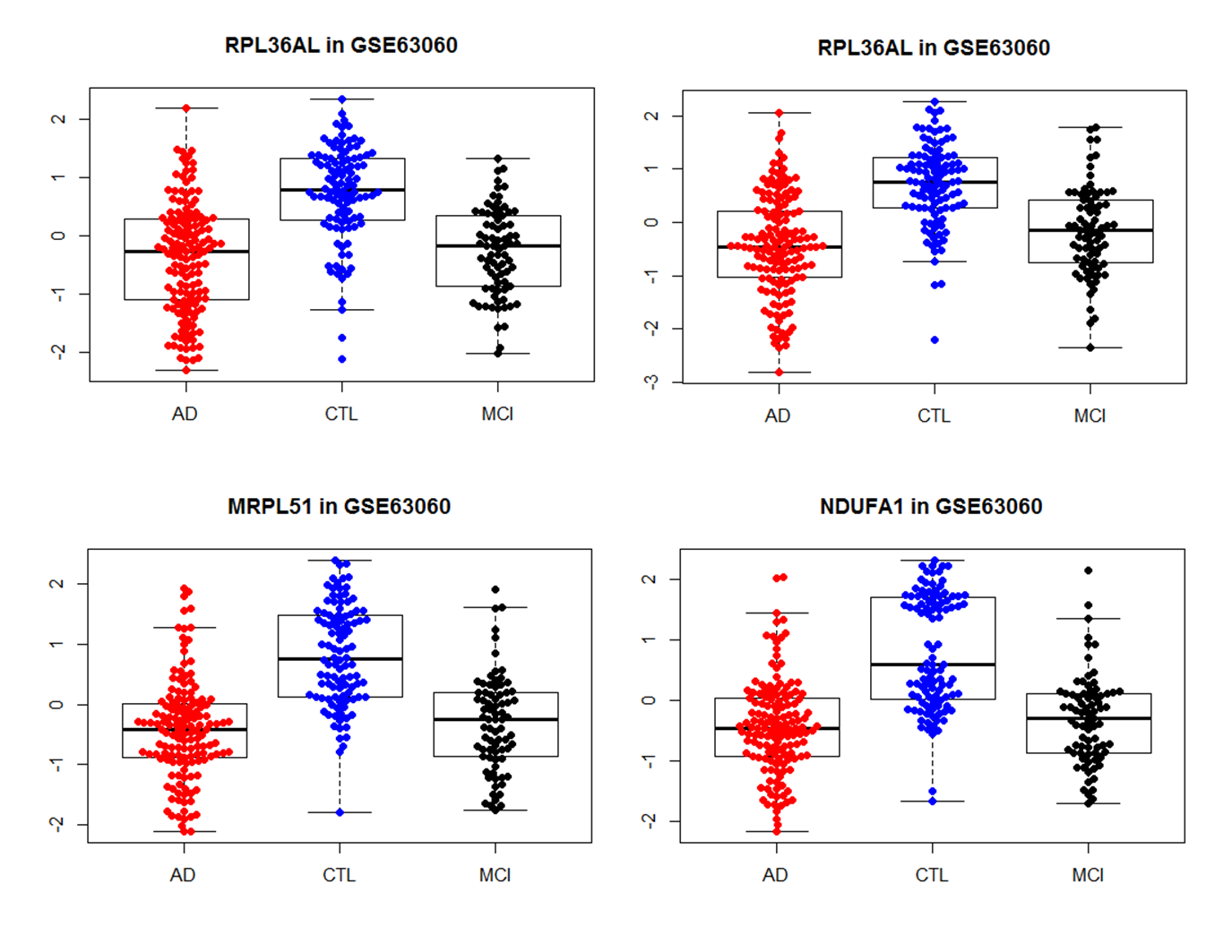
**

**
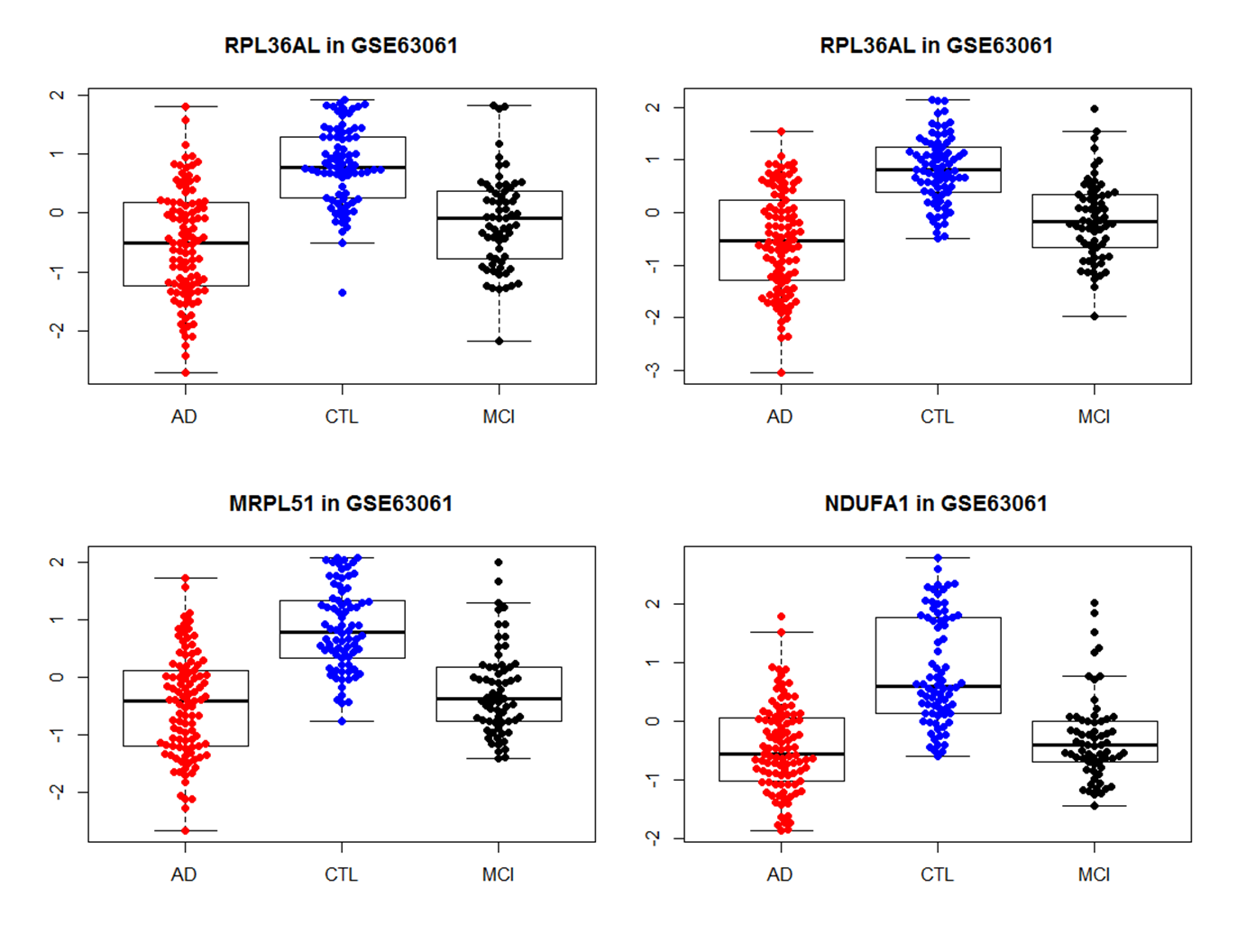
**

**Supplementary Figure 8. Precision-recall AUC for each case.** AUPR curves for SVM, RF and RR models using Full4set and Full6set for training features respectively. These values are reflected in Supplementary Table 6.

**
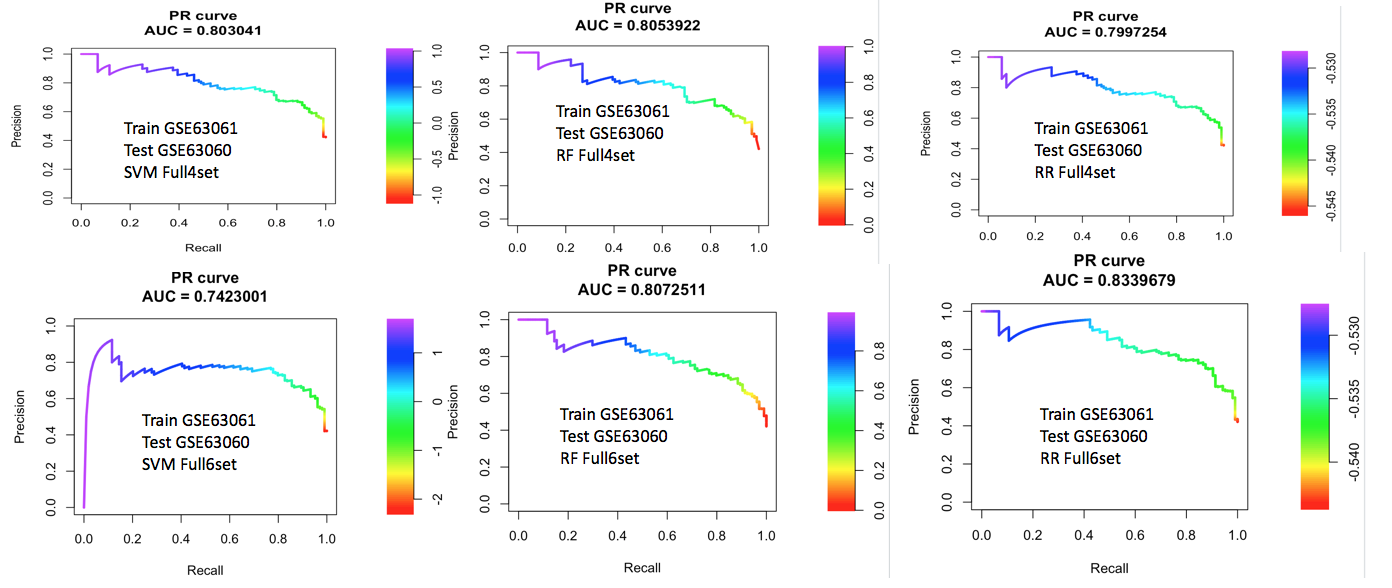
**

**
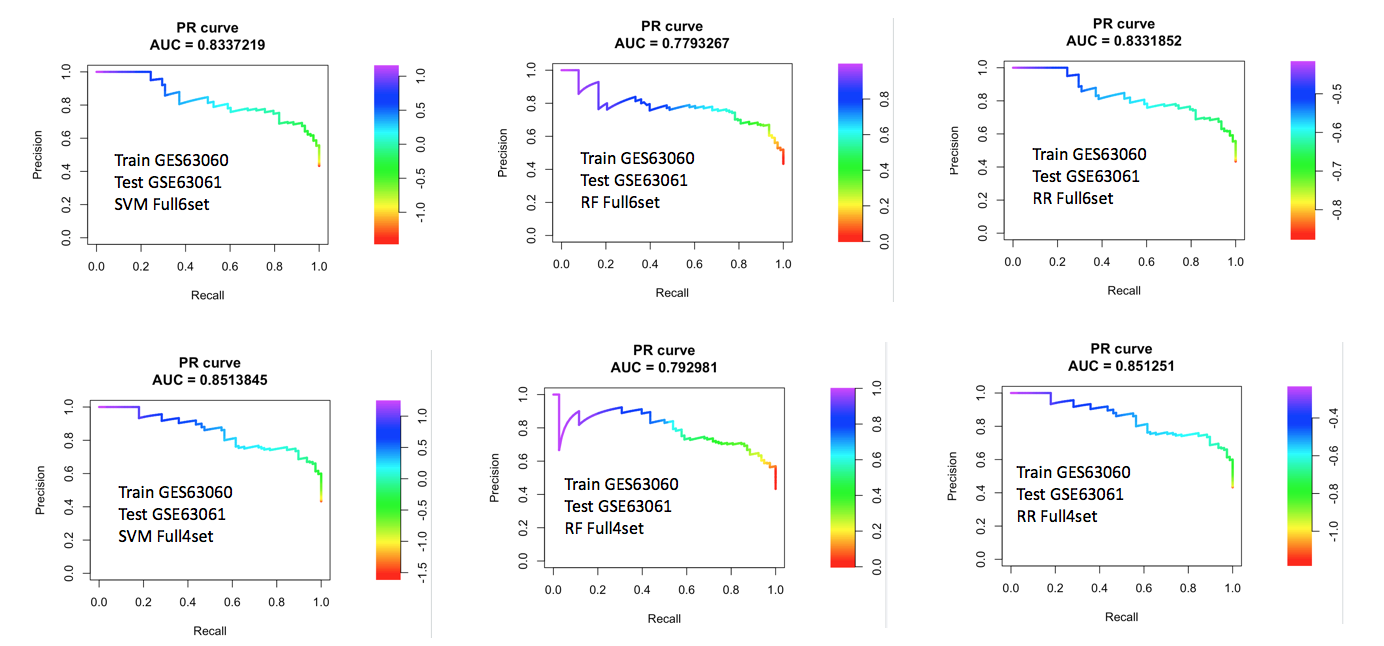
**

**Supplementary Table 1 Numbers of samples in different datasets. Meta-analysis refers to our previous gene expression analysis on brain tissues from the prefrontal cortex region (Li et.al, SREP, 2015).**

| **Dataset** | **Tissue** | **AD** | **MCI** | | **CTL** |
| --- | --- | --- | --- | --- | --- |
| GSE63060 | blood | 143 | 77 | | 104 |
| GSE63061 | blood | 102 | 65 | | 78 |
| GSE6613 | blood | 23 | 34 (early AD) | | 22 |
| ADNI2 | blood | **AD** | **EMCI** | **LMCI** | **CTL** |
|  | APOE4=0 | 10 | 57 | 36 | 91 |
|  | APOE4=1 | 22 | 28 | 32 | 31 |
|  | APOE4=2 | 6 | 5 | 9 | 4 |
| meta-analysis | brain | 450 |  | | 212 |
| GSE84422 | 19 Brain regions | **AD** |  |  | **CTL** |
|  | Anterior Cingulate (AC) | 16 |  |  | 11 |
|  | Caudate Nucleus (CN) | 16 |  |  | 8 |
|  | Dorsolateral Prefrontal Cortex (DPC) | 15 |  |  | 11 |
|  | Frontal Pole (FP) | 19 |  |  | 11 |
|  | Hippocampus (HIP) | 15 |  |  | 7 |
|  | Inferior Frontal Gyrus (IFG) | 15 |  |  | 8 |
|  | Inferior Temporal Gyrus (ITG) | 15 |  |  | 10 |
|  | Middle Temporal Gyrus (MTG) | 15 |  |  | 10 |
|  | Occipital Visual Cortex (OVC) | 10 |  |  | 9 |
|  | Parahippocampal Gyrus (PHG) | 19 |  |  | 10 |
|  | Posterior Cingulate Cortex (PCC) | 18 |  |  | 9 |
|  | Precentral Gyrus (PG) | 15 |  |  | 4 |
|  | Prefrontal Cortex (PC) | 17 |  |  | 7 |
|  | Putamen (PU) | 17 |  |  | 8 |
|  | Superior Parietal Lobule (SPL) | 10 |  |  | 9 |
|  | Superior Temporal Gyrus (STG) | 18 |  |  | 11 |
|  | Temporal Pole (TP) | 14 |  |  | 10 |
|  | Amygdala (AM) | 17 |  |  | 15 |
|  | Nucleus Accumbens (NA) | 17 |  |  | 13 |

**Supplementary Table 2 Excel file lists all DEGs in blood (AD and MCI).**

**Supplementary Table 3 Excel file lists IPA significant pathways for DEGs in blood.**

**Supplementary Table 4 Excel file lists DEGs identified in 19 brain regions.**

**Supplementary Table 5 Excel file lists IGAP MAGMA genes and their overlapped DEGs in blood and brain**.

**Supplementary Table 6.** **Classification performance of models with four or six features.** Here ACC Lower & Upper indicate the 95% interval of ACC; First column is AUC for ROC and Precision-recall value.

| Trained models with Full4set on GSE63061 | | | | | | | | | | |
| --- | --- | --- | --- | --- | --- | --- | --- | --- | --- | --- |
| Test data | AUROC/AUPR | Sens | Spec | PPV | NPV | ACC | Kappa | ACC Lower | ACC Upper | Method |
| GSE63060 | 0.86/0.803 | 0.755 | 0.798 | 0.837 | 0.703 | 0.773 | 0.543 | 0.716 | 0.824 | SVM |
| GSE63060 | 0.856/0.805 | 0.769 | 0.769 | 0.821 | 0.708 | 0.769 | 0.532 | 0.712 | 0.82 | RF |
| GSE63060 | 0.86/0.80 | 0.783 | 0.788 | 0.836 | 0.726 | 0.785 | 0.565 | 0.729 | 0.835 | RR |
| GSE63060 | 0.859/0.803 | 0.776 | 0.788 | 0.835 | 0.719 | 0.781 | 0.557 | 0.725 | 0.831 | Vote |
| Trained models with Full4set on GSE63060 | | |  |  |  |  |  |  |  |  |
| GSE63061 | 0.873/0.851 | 0.735 | 0.91 | 0.915 | 0.724 | 0.811 | 0.627 | 0.746 | 0.865 | SVM |
| GSE63061 | 0.861/0.793 | 0.804 | 0.718 | 0.788 | 0.737 | 0.767 | 0.523 | 0.698 | 0.826 | RF |
| GSE63061 | 0.89/0.851 | 0.775 | 0.846 | 0.868 | 0.742 | 0.806 | 0.611 | 0.74 | 0.861 | RR |
| GSE63061 | 0.875/0.832 | 0.765 | 0.872 | 0.886 | 0.739 | 0.811 | 0.623 | 0.746 | 0.865 | Vote |
| Trained models with Full6set on GSE63061 | | |  |  |  |  |  |  |  |  |
| GSE63060 | 0.851/0.742 | 0.755 | 0.827 | 0.857 | 0.711 | 0.785 | 0.569 | 0.729 | 0.835 | SVM |
| GSE63060 | 0.86/0.807 | 0.783 | 0.76 | 0.818 | 0.718 | 0.773 | 0.539 | 0.716 | 0.824 | RF |
| GSE63060 | 0.881/0.834 | 0.867 | 0.692 | 0.795 | 0.791 | 0.794 | 0.569 | 0.738 | 0.842 | RR |
| GSE63060 | 0.864/0.794 | 0.811 | 0.779 | 0.835 | 0.75 | 0.798 | 0.587 | 0.742 | 0.846 | Vote |
| Trained models with Full6set on GSE63060 | | |  |  |  |  |  |  |  |  |
| GSE63061 | 0.875/0.834 | 0.824 | 0.756 | 0.816 | 0.766 | 0.794 | 0.581 | 0.728 | 0.851 | SVM |
| GSE63061 | 0.849/0.779 | 0.735 | 0.795 | 0.824 | 0.697 | 0.761 | 0.522 | 0.692 | 0.821 | RF |
| GSE63061 | 0.874/0.833 | 0.706 | 0.885 | 0.889 | 0.697 | 0.783 | 0.572 | 0.716 | 0.841 | RR |
| GSE63061 | 0.866/0.815 | 0.755 | 0.821 | 0.846 | 0.719 | 0.783 | 0.566 | 0.716 | 0.841 | Vote |

**Supplementary Table 7. Prediction of MCI by using the trained models in AD vs. Control dataset.** The models trained in the dataset containing ADs and controls were used to test the dataset containing MCIs and Controls in the other cohort. 75.4% and 72.7% of MCI were predicted as AD in GSE63061 and GSE63060 respectively. Here ACC Lower & Upper indicate the 95% interval of ACC

| Trained AD/Ctr models in GSE63060 and tested MCI/Ctr in GSE63061 | | | | | | | | | | |
| --- | --- | --- | --- | --- | --- | --- | --- | --- | --- | --- |
| Method | AUC | Sensitivity | Specificity | PPV | NPV | Accuracy | Kappa | ACC Lower | ACC Upper | Features |
| SVM | 0.855 | 0.815 | 0.756 | 0.736 | 0.831 | 0.783 | 0.567 | 0.707 | 0.848 | Full6set |
| RF | 0.836 | 0.738 | 0.795 | 0.75 | 0.785 | 0.769 | 0.534 | 0.691 | 0.836 |
| RR | 0.856 | 0.708 | 0.885 | 0.836 | 0.784 | 0.804 | 0.6 | 0.73 | 0.866 |
| Vote | 0.849 | 0.754 | 0.821 | 0.778 | 0.8 | 0.79 | 0.576 | 0.714 | 0.854 |
| Trained AD/Ctr models in GSE63061 and tested MCI/Ctr in GSE63060 | | | | | | |  |  |  |  |
| SVM | 0.842 | 0.675 | 0.798 | 0.712 | 0.769 | 0.746 | 0.477 | 0.676 | 0.808 | Full4set |
| RF | 0.842 | 0.766 | 0.769 | 0.711 | 0.816 | 0.768 | 0.53 | 0.7 | 0.827 |
| RR | 0.842 | 0.74 | 0.788 | 0.722 | 0.804 | 0.768 | 0.527 | 0.7 | 0.827 |
| Vote | 0.842 | 0.727 | 0.788 | 0.718 | 0.796 | 0.762 | 0.515 | 0.694 | 0.822 |
